# Convolutional neural network to predict IDH mutation status in glioma from chemical exchange saturation transfer imaging at 7 Tesla

**DOI:** 10.3389/fonc.2023.1134626

**Published:** 2023-05-08

**Authors:** Yifan Yuan, Yang Yu, Jun Chang, Ying-Hua Chu, Wenwen Yu, Yi-Cheng Hsu, Liebig Alexander Patrick, Mianxin Liu, Qi Yue

**Affiliations:** ^1^ Department of Neurosurgery, Huashan Hospital, Shanghai Medical College, Fudan University, Shanghai, China; ^2^ National Center for Neurological Disorders, Shanghai, China; ^3^ Neurosurgical Institute of Fudan University, Shanghai, China; ^4^ Shanghai Clinical Medical Center of Neurosurgery, Shanghai, China; ^5^ Shanghai Key Laboratory of Brain Function Restoration and Neural Regeneration, Shanghai, China; ^6^ Research Units of New Technologies of Micro-Endoscopy Combination in Skull Base Surgery (2018RU008), Chinese Academy of Medical Sciences (CAMS), Shanghai, China; ^7^ Department of Radiology, Huashan Hospital, Shanghai Medical College, Fudan University, Shanghai, China; ^8^ Magnetic Resonance (MR) Collaboration, Siemens Healthineers Ltd., Shanghai, China; ^9^ Institute of Science and Technology for Brain-Inspired Intelligence, Fudan University, Shanghai, China; ^10^ Siemens Healthcare GmbH, Erlangen, Germany; ^11^ Shanghai Artificial Intelligence Laboratory, Shanghai, China

**Keywords:** convolutional neural network, chemical exchange saturation transfer, ultra-high field MR, radiomics, glioma

## Abstract

**Background and goal:**

Noninvasive prediction of isocitrate dehydrogenase (IDH) mutation status in glioma guides surgical strategies and individualized management. We explored the capability on preoperatively identifying IDH status of combining a convolutional neural network (CNN) and a novel imaging modality, ultra-high field 7.0 Tesla (T) chemical exchange saturation transfer (CEST) imaging.

**Method:**

We enrolled 84 glioma patients of different tumor grades in this retrospective study. Amide proton transfer CEST and structural Magnetic Resonance (MR) imaging at 7T were performed preoperatively, and the tumor regions are manually segmented, leading to the “annotation” maps that offers the location and shape information of the tumors. The tumor region slices in CEST and T1 images were further cropped out as samples and combined with the annotation maps, which were inputted to a 2D CNN model for generating IDH predictions. Further comparison analysis to radiomics-based prediction methods was performed to demonstrate the crucial role of CNN for predicting IDH based on CEST and T1 images.

**Results:**

A fivefold cross-validation was performed on the 84 patients and 4090 slices. We observed a model based on only CEST achieved accuracy of 74.01% ± 1.15%, and the area under the curve (AUC) of 0.8022 ± 0.0147. When using T1 image only, the prediction performances dropped to accuracy of 72.52% ± 1.12% and AUC of 0.7904 ± 0.0214, which indicates no superiority of CEST over T1. However, when we combined CEST and T1 together with the annotation maps, the performances of the CNN model were further boosted to accuracy of 82.94% ± 1.23% and AUC of 0.8868 ± 0.0055, suggesting the importance of a joint analysis of CEST and T1. Finally, using the same inputs, the CNN-based predictions achieved significantly improved performances above those from radiomics-based predictions (logistic regression and support vector machine) by 10% to 20% in all metrics.

**Conclusion:**

7T CEST and structural MRI jointly offer improved sensitivity and specificity of preoperative non-invasive imaging for the diagnosis of IDH mutation status. As the first study of CNN model on imaging acquired at ultra-high field MR, our results could demonstrate the potential of combining ultra-high-field CEST and CNN for facilitating decision-making in clinical practice. However, due to the limited cases and B1 inhomogeneities, the accuracy of this model will be improved in our further study.

## Introduction

Glioma is the most common primary intracranial tumor, with an incidence of about five to six per 100,000 people (1). Molecular pathology based on genetic testing has revolutionized the diagnosis of glioma and re-categorized the subtypes, thus providing more precious information for individualized therapy. Among various genetic markers, isocitrate dehydrogenase (IDH) mutation emerges as the most prominent one to predict chemosensitivity and influence overall survival (2). The precise identification of IDH mutation in pre-operative stage always leads to better diagnosis (3). Therefore, pre-operative identification of IDH status is highly valuable for neurosurgeons to formulate surgical strategy and decide the extent of tumor removal, especially for gliomas located at the eloquent brain area.

In recent years, a growing number of studies have started to investigate non-invasive prediction of IDH status by radiological imaging, especially using magnetic resonance imaging (MRI). Conventional T1-contrast imaging has been initially applied to differentiate high-grade gliomas from low-grade ones according to the appearance of enhanced signals, while later studies focusing on the non-enhancing area that proposed a T2-FLAIR mismatch sign have been applied to predict IDH mutation (3, 4). With diffusion-weighted imaging (DWI) becoming a routine at most institutions, some evidence has also emerged suggesting that DWI is promising for predicting IDH-mutation (5). Besides, the combination of deep learning and DWI for noninvasive classification of glioma genetic subtype was reported to be 5%–8.8% more accurate than anatomical imaging alone (6). However, studies based on structural Magnetic Resonance (MR) sequences ignored the close relevance of IDH to glioma metabolism and were not able to explain the underlying mechanism for prediction. To this extent, metabolic Magnetic Resonance (MR) imaging might play a more reasonable role in identifying IDH mutant glioma.

Chemical exchange saturation transfer (CEST) is a novel metabolic sequence to trace mobile proton exchange among water and other molecules. Triggering specific Magnetic Resonance (MR) signal at different radiofrequency pulses, it is prospective for assessing endogenous proteins and acidosis. Amide proton transfer (APT), a classical CEST approach that yields high contrast at 3.5 ppm frequency can theoretically detect elevated mobile peptides and proteins in gliomas and thus aid in the diagnosis. Jiang et al. has explored the APT-CEST manifestations of IDH mutant glioma and found that APTw imaging hyperintensity could be a potential marker of active malignant glioma and is able to distinguish between regions of heterogeneous abnormality on anatomical brain MRI with 85.1% sensitivity and 94.1% specificity (7). In addition, introduction of ultra-high-field to CEST imaging recently is making its stratification of glioma more sufficient (8). Our group previously reported that IDH wild-type cases generated higher APT values than mutant cases, indicating potential ability of CEST to differentiate IDH status (9).

With the rapid development of medical image analysis in the past decade, image-based radiomics and deep learning are serving as indispensable tools to determine genetic biomarkers using imaging features. Among them, deep convolutional neural network (CNN) is a representative method to automatically exploit high-dimensional information from images by learning to identify predictive features under supervisions, while image-based radiomics requires designs of hand-crafted features. In addition, the deep-layer model could tackle the potentially highly non-linear relationship between the extracted feature and the tumor property to be predicted and, thus, could be more proper tools to improve predictive accuracy and has been used to identify molecular markers in glioma. However, CNN has not been utilized in 7T CEST to predict IDH status in glioma. In this study, we, for the first-time, study a CNN model based on ultra-high field T1 and CEST combination to estimate the IDH-mutated status and compare it with radiomics-based methods. We hypothesized that the multi-modal based deep learning algorithm can achieve high accuracy in noninvasively stratification of glioma than single-modal based deep learning or conventional radiomics-based methods.

## Method and materials

### Participants

This retrospective study was approved by the local institutional review board, and the requirement to obtain informed consent was waived. Patients were recruited from Huashan Hospital, Fudan University between August 2020 and September 2022. All patients were newly diagnosed as glioma and underwent subsequent resection or biopsy. Pathologic diagnoses were determined according to the 2021 World Health Organization (WHO) classification of central nervous system (CNS) tumors, and patients diagnosed with a non-glioma disease were excluded. The study was registered in WHO ICTRP (registration No. ChiCTR2000036816) and 84 patients were included.

### MRI and IDH1 type acquisition

All patients underwent MRI scan within a week prior to surgery. CEST MRI was performed on a 7T MRI scanner (MAGNETOM Terra; Siemens Healthineers, Erlangen, Germany) with a prototype-developed snapshot‐CEST sequence based on a 3D gradient spoiled GRE readout (10) with a single-channel transmit/32-channel receive head coil (Nova Medical, Wilmington, MA, USA). The snapshot‐CEST sequence parameters were TR = 3.4 ms, TE = 1.59 ms, FA = 6°, resolution = 1.6 mm × 1.6 mm × 5 mm, and GRAPPA acceleration factor = 3 with amplitudes B1 = 0.6, 0.75, and 0.9 mT. Z-spectra were sampled unevenly by 56 frequency offsets between -300 ppm and +300 ppm. The Z‐spectrum data were corrected for both B0 and B1 inhomogeneities using the WASABI method (11) and were fit pixel-wise by a five-pool Lorentzian model (water, amide, amine, NOE, and MT) using the Levenberg–Marquardt algorithm (12). For CEST data co-registration, high-resolution T1 MP2RAGE (TR = 3800 ms, TI1 = 800 ms, TI2 = 2700 ms, TE = 2.29 ms, FA = 7°, and resolution = 0.7 mm isotropic) and 3D T2-SPACE (TR = 4000 ms, TE = 118 ms, and resolution = 0.67 mm isotropic) were acquired at 7T. Routine-clinical-sequence, contrast-enhanced T1-weighted images (TR = 6.49 ms, TE = 2.9ms, FA = 8°, spatial resolution = 0.833 mm × 0.833 mm × 1 mm), were acquired at 3T on an Ingenia MRI scanner (Koninklijke Philips N.V., Netherlands). The MRI data were processed by experienced imaging engineers using MATLAB (R2020a, USA).

Tumor tissues obtained during operations were collected for histological analysis. IDH mutation status was evaluated by next-generation sequencing of IDH1 and IDH2 genes or by IHC (anti-IDH1 antibody, ab172964, Abcam, American) using standard techniques.

### Data preprocessing

An experienced radiologist manually annotated the tumor regions. The annotation was delineated based on the CE images (high grade gliomas) and T2 images (low grade gliomas). Then, annotation was co-registered to the T1 structure images and CEST images required at 7T. The intensity of each participant’s CEST and T1 image was a z-score normalized over the whole volume. Each selected CEST or T1 slice included more than 100 pixels of tumor regions. All slices were further cropped according to the tumor region bounding box and re-sized into 100 × 100 pixels. Each pair consisted of one CEST slice, one T1 slice, and one tumor-annotated mask slice with an IDH label, and a total of 4093 pairs were selected as our training and testing datasets.

### Convolutional neural network

**Figure 1:**
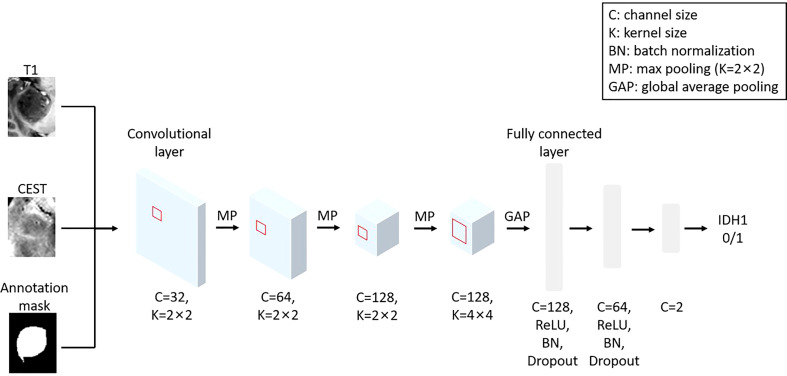
The architecture of the implemented CNN model using T1, CEST and annotation mask as inputs.

A 2D convolutional neural network (CNN) was designed to generate IDH mutation predictions. The preprocessed CEST, T1, and tumor annotation slices were combined as three channels and input into the CNN. Under this setting, our model combined the metabolism information from CEST, the anatomical information from T1, and the enhanced shape information from the tumor annotation for the IDH prediction. The architecture and the parameter settings are depicted in **Figure 1**.

We train the CNN with training epoch = 35, learning rate = 0.01 for first 10 epochs, and 0.001 for the 10^th^ to 20^th^ epochs and 0.0001 for the remaining epochs, and batch size = 32. The “Adam” algorithm (13) was used to automatically optimize the trainable parameters with a weight decay of 0.001. In **Figure S1**, it can be observed that the loss function value and accuracy in the training set of our CNN model can quickly converge to a static level near 0.5 after 15–20 training epochs, suggesting our model can fast-extract the informative feature from the Magnetic Resonance (MR) data and our training epoch length (i.e. epoch = 35) can guarantee the final prediction is from a set of relatively stable parameters.

We adopted a standard hierarchical design of the CNN. In the first three convolutional layers, we implemented a convolution kernel with 2×2 pixel^2^ to extract local features and max pool to refine the spatial information. Along with the reduction of image spatial size, we increase the size of feature channels to maintain (or increase) the amount of information. In the fourth layer, the elements in the feature map have become representatives of each local region. At this stage, we implemented a larger kernel (4×4 pixel^2^) to allow cross-talks of information in a larger spatial scale, without changing in the channel size. All elements in the feature map will be integrated by a global average pooling. The global feature will then be sent into three layers of full-connected layers (attached with non-linear activation function “ReLU”, batch normalization and dropout) for generating the final prediction.

Since the sample size was slightly imbalanced in different groups, the weighted cross-entropy was employed as the loss function, with weights adaptively configured according to the ratio in sample sizes between wild-type and mutant groups. Other parameters of the neural network models are initialized with random weights. The training is accelerated with an Nvidia GTX 3080 GPU.

### Radiomics

The open-source toolbox “Pyradiomics” [https://pyradiomics.readthedocs.io/en/latest/ (14)] was implemented to compute radiomics features from the T1, CEST, and annotation slices. The extracted radiomics features included 2D shapes, first order statistics, gray level co-occurrence matrix, gray level run length matrix, gray level size zone matrix, neighboring gray tone difference matrix, and gray level dependence matrix. By design, the annotation mask was required to compute 2D shapes and first order statistics and thus in all radiomics-based predictions the annotation mask was used as inputs. In total, 95 features were computed from each slice when using “T1 + annotation” or “CEST + annotation” as inputs and 188 features when using “T1 + CEST + annotation” as inputs. The resulted features were then fed into a conventional machine learning classifier. Results from two methods were presented in the main text: 1) a linear logistic regression (LR) model with L1-penalty for classification, which hypothesizes the sparsity in the features; and 2) a non-linear support vector machine (SVM) with radial basis function (RBF) kernel. The LR and SVM methods were implemented with the open-source “scikit-learn” toolbox. Results from LR and SVM under other configurations, such as applying L2-penalty for LR and other kernels for SVM, can be found in **Tables S1** and **S2**. To address the imbalance of the sample size in the two classes, weights were automatically set on the classes according to the inverse proportion of class frequencies in the training data. Other parameters were set to be default configurations.

### Validation scheme

A five-fold cross-validation was performed to assess the predictability of our method. The 4693 slices were randomly split into five equal folds, with four folds being the training set and the remaining one being the testing set. Five rounds of validations were performed so that each fold behaved as testing set once. For predictions from each round, we computed four metrics from different aspects to evaluate the performance, i.e., the accuracy (ACC), sensitivity (SEN), specificity (SPE), and area under the receiver operating characteristic curve (AUC).

### Statistical analysis

The means, standard deviation (STD) and 95% confidence interval (CI) of the performance metrics from cross-validation were calculated and reported. The comparisons on the performance metrics of different methods from cross-validation were performed using one-sided paired *t*-test.

In addition, we also integrated the predictions from each fold to compute the metrics on predictions for all the participants. The significance of this integrated result was estimated by a permutation test. The performance metrics were re-calculated using the CNN model predictions and randomly permutated ground truth labels. Such setting mimics the performances of null models under chance level. With 1000 times of permutation, the distribution of the performance metrics under chance level was constructed. The p-values were obtained by the probability of finding a metrics value that was larger than the real metrics value from CNN in the chance level distribution.

We also constructed the AUCs based on the integrated prediction from different experimental conditions and use the Delong’s test to verify the differences among the AUCs (15).

All statistical analyses were performed in Matlab (R2022b).

## Results

### Characteristics of the studied population

The patient demographics were summarized in **Table 1**. Eighty-four patients were enrolled in the study, including 22 oligodendrocytomas, 28 astrocytomas, 30 glioblastomas, one diffuse midline glioma, one ganglioglioma, and two pediatric high-grade gliomas. Almost half of the tumors were located in the frontal lobe, followed by the temporal and insular lobes. The detailed distribution is visualized in **Figure S2**. Among them, 44 were IDH wild-type. Patients of WHO grade 2 and grade 3 were all IDH mutant; while two grade 4 patients were IDH mutant. There was no significant difference between IDH wild-type and mutant groups in gender and handedness. However, the mean age was significantly lower in the mutant group (p = 0.0026). Interestingly, five IDH wild-type gliomas, which tended to be WHO grade 3 in morphology, were categorized as WHO grade 4 based on the 2021 WHO neuro-oncology classification.

**Table 1:** The demographic and tumor-related characteristics of the included patients.

	All Patients	WHO	WHO	WHO	WHO
		Grade 1	Grade 2	Grade 3	Grade 4
No. of patients					
	84	1	27	11	45
Age (yr)					
Mean	48.8	72	42.1	45.1	57.4
Range	25-75	/	25-65	29-67	36-75
Gender					
Male	45	0	12	7	26
Female	39	1	15	4	19
Positon					
Frontal Lobe	34	0	16	5	13
Parietial Lobe	6	0	2	0	4
Occipital Lobe	4	0	0	0	4
Temporal & Insular Lobe	32	0	8	6	18
Others	8	1	1	0	6
IDH status					
Wild-type	44	1	0	0	43
Mutant	40	0	27	11	2

**Figure 2:**
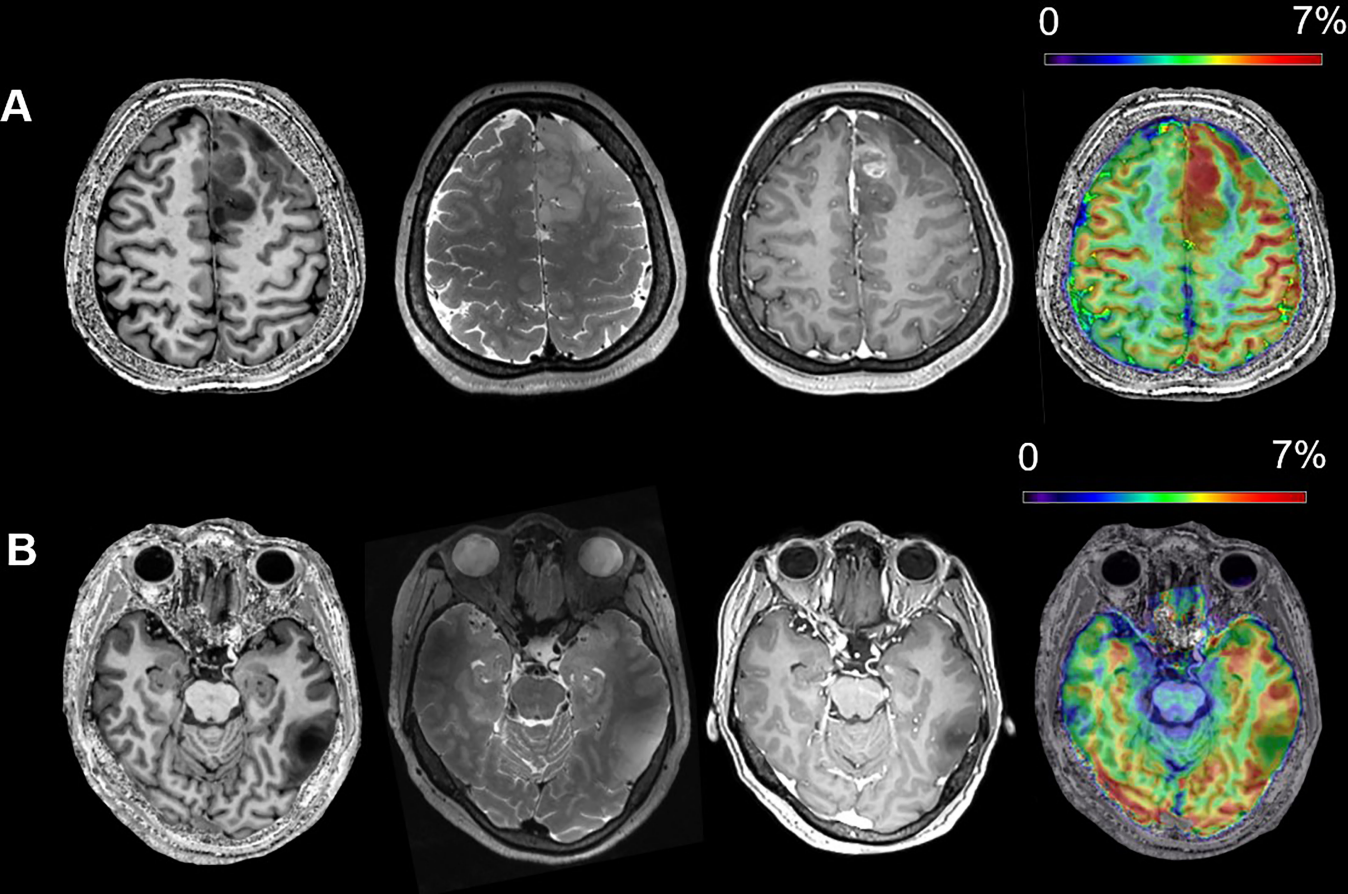
The typical MR appearances of an IDH wild-type **(A)** and an IDH mutant **(B)** glioma (from left to right, T1WI, T2WI, Gadolinium Enhanced T1WI and APT). **(A)** A 40-year-old female patient with IDH wild-type glioblastoma, WHO grade 4; **(B)** a 42-year-old female patient with IDH mutant astrocytoma, WHO grade 2.

In **Figure 2**, we visualize the typical appearances for IDH mutant and wild-type gliomas in conventional Magnetic Resonance (MR) and CEST. **Figure 2A** was derived from a 40-year-old female patient who suffered from intermediate headache and post-surgical pathology indicated that the right fontal lesion was an IDH wild-type GBM (glioblastoma, WHO grade 4). On the other hand, lesion in **Figure 2B** was a left temporal low-grade glioma from a 42-year-old female and she was diagnosed as IDH mutant astrocytoma (WHO grade 2). It can be observed that the APT% in the lesion of IDH wild-type (**Figure 2A**) is much higher than which of IDH mutant lesion (**Figure 2B**). These visualizable differences in the APT-CEST imaging data provide the basis for our further deep learning method building for accurate IDH predictions.

### IDH predictions using CNN based on T1, T2, and CEST

**Figure 3:**
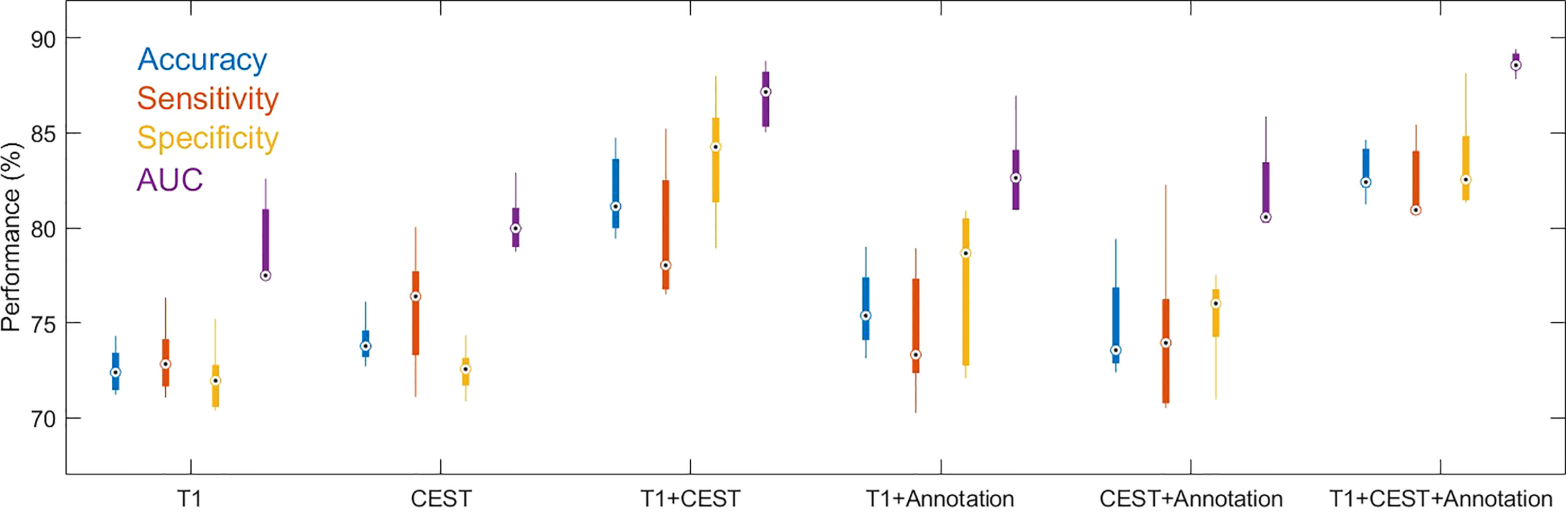
Boxplots for the distributions of performance metrics from the cross-validations out of models using different inputs. The lower and upper ends of the box body indicate the first and the third quantiles, and the lower and upper ends of the whiskers indicate the minimum and maximum values. The black dot with circle suggested the median.

**Figure 4:**
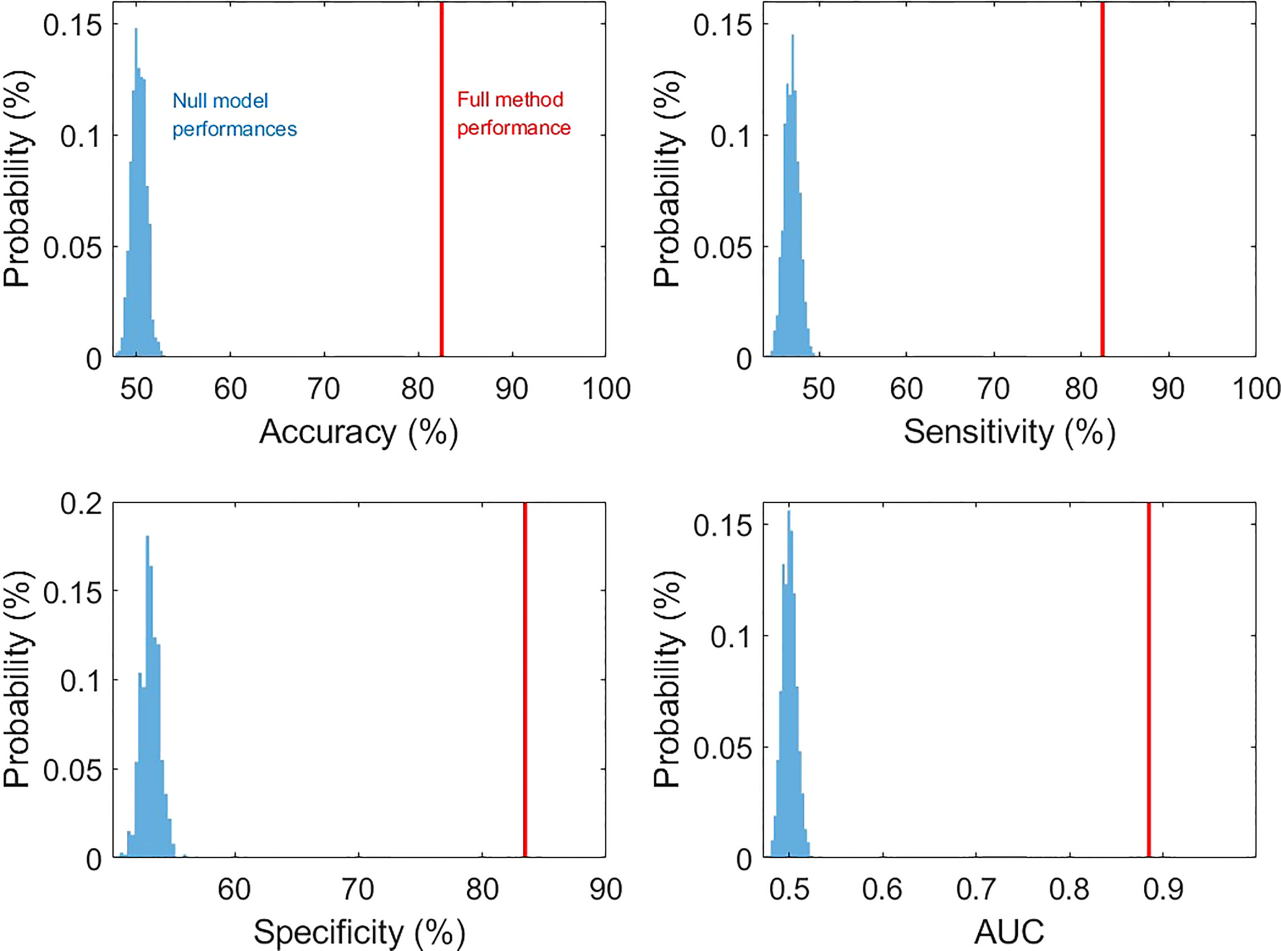
The comparison of prediction metric values from our method and those from null models (permutation tests with 1000 times of realizations).

In the cross-validations, our full method using T1, CEST, and annotation mask as inputs (**Table 2** and **Figure 3**, “T1+CEST+annotation”) obtained ACC = 82.94% (CI = [81.24%, 84.65%]), SEN = 82.35% (CI = [79.76%, 84.94%]), SPE = 83.45 (CI = [79.98%, 86.92%]), and AUC = 0.8868 (CI = [0.8792, 0.8944]). When integrating the predictions, the metrics values were ACC = 82.94% (p < 0.001), SEN = 82.39% (p < 0.001), SPE = 83.45 (p < 0.001), and AUC = 0.8849 (p < 0.001), all of which showed significance when comparing to the chance levels (**Figure 4**).

**Table 2:** CNN-based prediction performances under models using different inputs.

Input	ACC (%)	SEN (%)	SPE (%)	AUC
T1	72.52 ± 1.12***	73.10 ± 1.80**	72.03 ± 1.72***	0.7904 ± 0.0214***
CEST	74.01 ± 1.15***	75.70 ± 3.00*	72.50 ± 1.14***	0.8022 ± 0.0147***
T1 + CEST	81.75 ± 1.98	79.64 ± 3.33	83.68 ± 3.03	0.8689 ± 0.0145*
T1 + annotation	75.76 ± 2.02**	74.47 ± 3.05**	76.70 ± 3.74*	0.8293 ± 0.0221**
CEST + annotation	74.88 ± 2.58**	74.37 ± 4.24**	75.28 ± 2.27*	0.8192 ± 0.0216**
T1 + CEST + annotation	82.94 ± 1.23	82.35 ± 1.87	83.45 ± 2.50	0.8868 ± 0.0055

The data are reported in form of “Mean ± STD”. * “CEST + T1 + annotation” is significantly higher than the indicated method at p *<* 0.05 level. ** p *<* 0.01. *** p *<* 0.001.

### Ablation study

The ablation analysis in the inputs for our method was conducted to address the contributions of each modality. We trained the CNN using only CEST image (**Table 2** and **Figure 3**, “CEST”). When using CEST only, the prediction performances dropped to ACC = 74.01% (CI = [72.41%, 75.61%]), SEN = 75.70% (CI = [71.54%, 79.86%]), SPE = 72.50% (CI = [70.91%, 74.09%]), and AUC = 0.8022 (CI = [0.7818, 0.8227]). Besides, we built another model based on T1 image data only (**Table 2** and **Figure 3**, “T1”). It can be observed that the performance metrics from the model based on T1 image were ACC = 72.52% (CI = [70.96%, 74.07%]), SEN = 73.10% (CI = [70.60%, 75.60%), SPE = 72.03% (CI = [69.64%, 74.42%]), and AUC = 0.7904 (CI = [0.7607, 0.8201]), which were slightly lower than those based on CEST (**Table 2** and **Figure 3**. p = 0.1136 for ACC, p = 0.1502 for SEN, p = 0.2162 for SPE, p = 0.2316 for AUC; one-sided paired *t*-test). Note that these metrics were both higher than the chance level (see null model distributions in **Figure 4**). It suggested that using CEST and T1 image only can still provide satisfactory predictability on the IDH1 mutation. Besides, CEST image exhibited a slight advantage over T1 image (no statistical significance is identified).

Similarly, we tried models based on T1 and T2 (standard T2, due to data availability) and compared it with the models based on T1 and CEST. If there is no significant difference, the improvements of adding CEST indeed originates from T2 weighting. After aligning the data, we selected 74 subjects and obtained 4096 slices to perform the experiment and comparison. In the results of **Table 3**, we observed that with or without the annotation, the models based on T1 + CEST can both significantly outperform the models based on T1 + T2. Therefore, the metabolism information from CEST, besides the T2-weighting information, is providing additional assistance to the prediction.

**Table 3:** The prediction performances of models based on T1 + T2 and T1 + CEST.

Input	ACC (%)	SEN (%)	SPE (%)	AUC
T1 + CEST	80.03 ± 0.18**	82.45 ± 2.55***	77.65 ± 4.97	0.8553 ± 0.0152*
T1 + T2	75.59 ± 1.82	74.77 ± 2.21	76.38 ± 3.48	0.8193 ± 0.0218
T1 + CEST + annotation	82.25 ± 1.57**	83.36 ± 4.05*	81.15 ± 4.08	0.8839 ± 0.0181*
T1 + T2 + annotation	77.30 ± 2.40	75.89 ± 5.48	78.53 ± 4.48	0.8496 ± 0.0219

“*” indicates significant improvements of the models based on T1 + CEST over T1 + T2 at p < 0.05 level, based on one-sided paired *t*-test. **p<0.01; ***p<0.001.

Besides CNN developed from single modality, the efficacy of multi-modal combination was further tested. When using both modalities as inputs (without the annotation mask, “T1 + CEST”), the CNN model achieved ACC = 81.75% (CI = [79.00%, 84.50%]), SEN = 79.64% (CI = [75.02%, 84.26%]), SPE = 83.68% (CI = [79.47%, 87.89%]) and AUC = 0.8689 (CI = [0.8487, 0.8891]), which were significantly higher than the corresponding results from both single-modal-based predictions (**Table 2** and **Figure 3**, “T1” and “CEST”). When comparing “T1” and “T1 + CEST”, one-sided *t*-test yielded p = 0.0002 for ACC, p = 0.0048 for SEN, p = 0.0005 for SPE and p = 0.0002 for AUC. When comparing “CEST” and “T1+CEST”, one-sided *t*-test yielded p = 0.0011 for ACC, p = 0.1319 for SEN, p = 0.0010 for SPE, and p = 0.0005 for AUC. These results supported the advantage of multi-modal information fusion.

In addition to T1 and CEST modalities, the geometrical information (i.e. shape of the tumor) from the annotation mask could be optimally utilized by the CNN. When adding the annotation mask as one of the inputs (**Table 2** and **Figure 3**, “T1 + annotation”, “CEST + annotation”, “T1 + CEST + annotation”), the model provided higher performances than their corresponding versions that without the annotation mask (**Table 2**, “T1”, “CEST”, “T1 + CEST”). When comparing “T1” and “T1 + annotation”, one-sided *t*-test yielded p = 0.0180 for ACC, p = 0.2297 for SEN, p = 0.0301 for SPE and p = 0.0287 for AUC. When comparing “CEST” and “CEST + annotation”, one-sided *t*-test yielded p = 0.3180 for ACC, p = 0.6378 for SEN, p = 0.0291 for SPE, and p = 0.1565 for AUC. When comparing “T1 + CEST” and “T1 + CEST + annotation”, one-sided *t*-test yielded p = 0.1300 for ACC, p = 0.0618 for SEN, p = 0.5631 for SPE, and p = 0.0120 for AUC. These observations highlighted the annotation mask could enhance the tumor region to assist other modality for better predictions.

**Figure 5:**
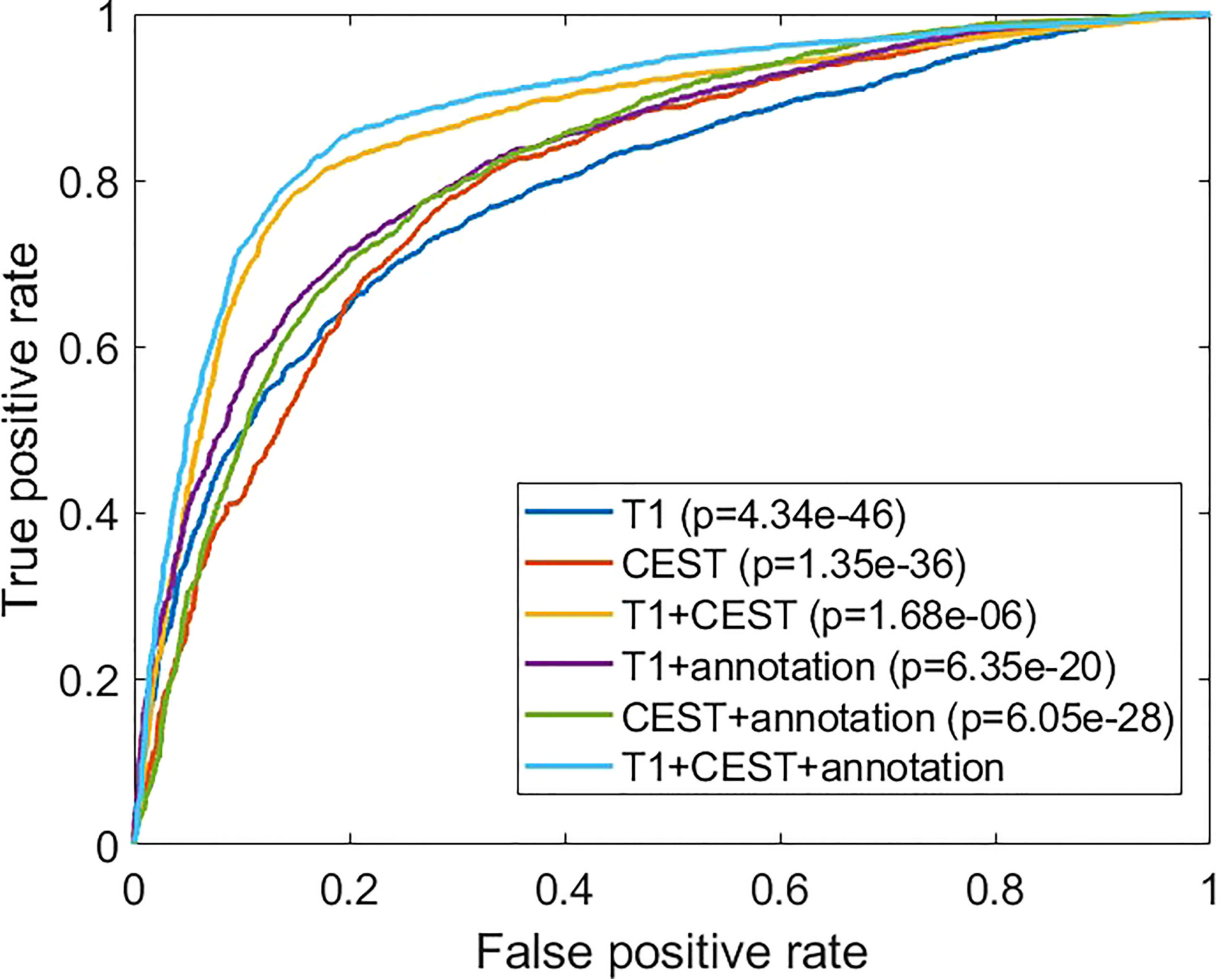
Comparisons of the receiver operating characteristic curves from different experimental conditions. The *p-*values are from comparison between the indicated method with the full method (“T1 + CEST + annotation”) using Delong’s test.

In **Figure 5**, we analyzed the receiver-operating characteristic (ROC) curves from models with different inputs. In general, the results were consistent to the observations in **Table 2**. Our full method also showed strong significances over all compared settings in terms of the ROC curve evidenced by the Delong’s tests.

### Comparison between CNN and radiomics

Considering widely application of radiomics in gene prediction, further analysis was performed to compare the CNN-based prediction methods and radiomics-based prediction methods. In **Tables 3**, **S1**, and **S2**, we firstly investigated the results by applying different machine-learning based classification methods on the radiomics features under different configurations. Overall, it can be found that the predictability of radiomics-based methods varies remarkably across different methodological settings. Taking the “T1 + CEST + annotation” in **Table 3** for example, logistic regression based radiomics achieved ACC = 76.35%, SEN = 77.95%, SPE = 74.89%, and AUC = 0.7641, while SVM based radiomics achieved ACC = 64.13%, SEN = 66.30%, SPE = 62.11%, and AUC = 0.6420. Therefore, the performances of radiomics-based predictions can be strongly depending on and restricted by the method selections. Secondly, in all the methods (**Tables 4** and **S1**, **S2**), it can be consistently observed that using CEST as inputs can lead to better performances than using T1; and using both CEST and T1 as inputs results in higher predictability than using a single modality. In addition, in **Table S1**, the L1-penalty optimizes the prediction results under LR method, which suggests the sparsity of the predictive radiomics features. In parallel, within SVM methods, the non-linear kernels generally improve the performances, indicating the non-linearity of the relationship between the radiomics feature and the IDH mutation. However, none of the SVM achieves performances being higher than results from LR, which may be due to the limited classification capacity of the SVM model.

**Table 4:** Comparison between CNN-based predictions and radiomics-based predictions with different inputs (Mean ± STD).

Method	Input	ACC (%)	SEN (%)	SPE (%)	AUC
	T1 + annotation	75.76 ± 2.02	74.47 ± 3.05	76.70 ± 3.74	0.8293 ± 0.0221
CNN	CEST + annotation	74.88 ± 2.58	74.37 ± 4.24	75.28 ± 2.27	0.8192 ± 0.0216
	CEST + T1 + annotation	82.94 ± 1.23	82.34 ± 1.87	83.45 ± 2.50	0.8868 ± 0.0055
Rad +LR +L1	T1 + annotation	67.36 ± 1.70***	67.16 ± 2.35**	67.57 ± 1.81**	0.6736 ± 0.0170***
	CEST + annotation	72.03 ± 1.37*	77.51 ± 3.82	66.89 ± 2.68**	0.7220 ± 0.0151***
	CEST + T1 + annotation	76.35 ± 1.98**	77.95 ± 3.04*	74.89 ± 1.92**	0.7641 ± 0.0204***
Rad +SVM +RBF	T1 + annotation	54.80 ± 1.23***	68.83 ± 4.04***	41.92 ± 1.53***	0.5537 ± 0.0156***
	CEST + annotation	58.08 ± 10.1***	83.30 ± 3.00***	34.68 ± 2.6***	0.5899 ± 0.0082***
	CEST + T1 + annotation	64.13 ± 1.91***	66.30 ± 2.61***	62.11 ± 1.73***	0.6420 ± 0.0194***

*DL-based model is significantly better than the radiomics-based method using same inputs at p *<* 0.05 level. **]p *<* 0.01. ***]p *<* 0.001. Rad, Radiomics; LR, logistic regression; SVM, support vector machine; L1, L1-penalty; RBF, radial basis function kernel.

**Figure 6:**
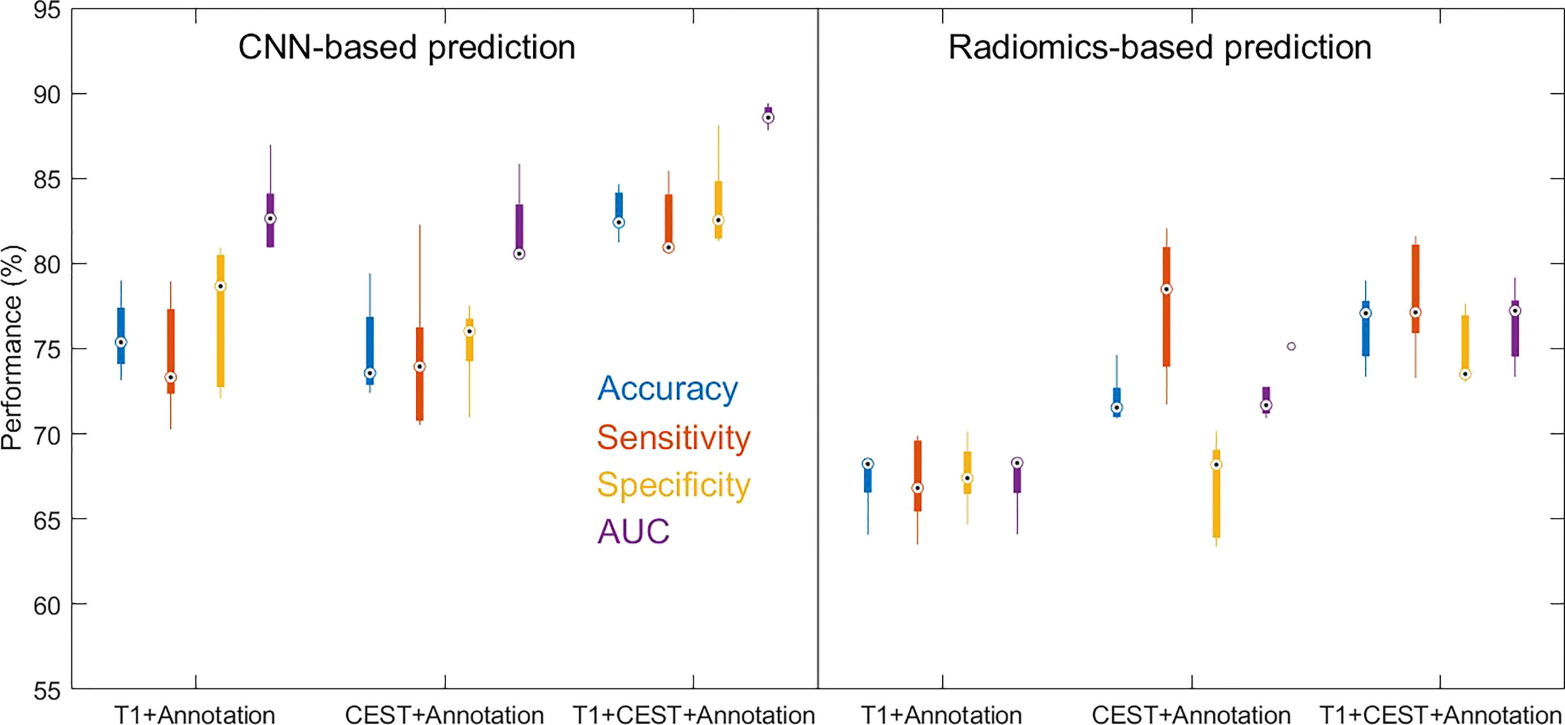
Boxplots for the distributions of performance metrics from the cross-validations out of CNN-based prediction method and radiomics-based prediction method (with logistic regression as classifier) using different inputs.

We further compare the radiomics-based methods with CNN. In the results in **Table 3** and **Figure 6**, we observed that in all three input settings, the CNN-based predictions achieved remarkably improved performances above those from radiomics-based predictions by 10% to 20% in all metrics, with strong statistical significance indicated. For logistic regression, the performances range from 67% to 76% while the SVM never achieves results above 65%. In comparison, the CNN yielded strikingly superior metrics, with accuracy from 74.88% to 82.94% and AUC from 0.8192 to 0.8868. These results directly suggested the advantage of CNN based methods over radiomics-based methods.

## Discussion

IDH mutation is by far the most critical gene mutation in the diagnosis of glioma, suggesting good chemosensitivity and better prognosis, and therefore in turn can influence surgical decisions. With the thriving development of radiogenomics, a growing number of studies are being conducted to reveal the imaging features of IDH-mutant gliomas and thus enable noninvasive prediction. In this study, we innovated from ultra-high-field MRI, CEST metabolic sequences, and multimodal combination to construct a novel CNN prediction model. The model predicts IDH mutation in glioma with an accuracy of 82.94% and outperforms a radiomics approach, offering promise for preoperative noninvasive molecular diagnosis and even surgical decision-making.

The vigorous development of gene sequencing technology in the past decade has promoted the understanding of the molecular mechanism of glioma at multiple levels such as DNA, RNA, and epigenetics. These genetic characteristics such as IDH mutation correspond to distinct prognosis and individualized therapy, and therefore were enrolled in the WHO 2021 version of the central nervous system oncology classification. However, there still remain several drawbacks in clinical practice for molecular stratification. First, the procedure to obtain samples for gene testing was invasive *via* stereotaxic biopsy or tumor resection. Second, since the tested specimens can only be taken from a portion of the tumor, sampling bias is almost inevitable given the spatial heterogeneity of the genes. Third, considering the huge gap of technical level and economic development among different centers, the cost for gene sequencing is still high and not covered by most insurance. Thus, a new method which may cover the “invasiveness”, “sampling bias”, and “high cost” can further promote the precise diagnosis and treatment of glioma. Empowering imaging, especially MRI, to predict molecular features of tumors through more advanced techniques or algorithms is the most promising means to address these issues. On the one hand, it can enable non-invasive preoperative diagnosis, and on the other hand, it is prospered to portray the full genetic picture of tumors in three dimensions.

Unlike the majority of previous studies focused on structural imaging, this study attempted to implement the metabolic sequence CEST for IDH prediction. APT-CEST can indirectly reflect the content of mobile peptides and endogenous proteins through semi-quantitative measurement of amide bonds, and therefore exhibit high signal in malignant tumors. It has been demonstrated that it traces glioma hypermetabolic regions at good agreement with amino acid PET. Given the correlation between IDH mutation and tumor malignancy, we hypothesized that CEST might implicitly suggest IDH status by monitoring the degree of tumor metabolism. To this end, we preliminarily investigated the role of CEST in discriminating gliomas with different IDH status and identified a lower signal in the IDH mutant subgroup. Similar findings have been reported by other research groups. However, there is a lack of IDH prediction models constructed directly based on CEST. In this paper, we constructed CNN models from different modalities and revealed that the sensitivity and specificity of CEST alone were slightly better than T1, and the efficacy was significantly improved after combining the two. Such observation was confirmed with the radiomics-based analyses. This result confirmed that CEST, as a novel metabolic sequence, may have superiority over conventional structural images for metabolic characterization such as IDH mutation. Nevertheless, whether IDH mutation can mechanically cause the distinctive manifestation on CEST remains to be further investigated.

All imaging data in this study were acquired from 7T ultra-high field. On T1 structural images, 7T will provide higher spatial resolution, more detailed intracranial anatomy and clearer lesion features compared to 3T. For CEST imaging, the advantages of ultra-high field strength are even more pronounced in terms of higher chemical transfer separation, better signal-to-noise ratio, and 3D multilayer scanning. In addition, some low concentrations of amide protons which cannot be captured at 3T will be properly detected at 7T. For these reasons, 7T should theoretically predict IDH mutations better than 3T. Paech et al. used relaxation–compensated multipool CEST at 7T and found that the AUC for predicting IDH mutation was as high as 91.84% (8). However, considering that only 31 patients were included, the generalizability of their findings needs to be evaluated with caution. Our study also did not set up a control group at 3T, so the pros and cons of the two could not be directly compared. Previous studies using 7T to predict IDH mutation have mostly relied on MRS (MR spectroscopy) to detect its metabolite 2-hydroxyglutarate (2-HG). Berrington et al. compared the effect of using MRS to detect 2-HG in phantoms and patients at different field strengths and confirmed that 7T significantly enhanced the sensitivity for 2-HG detection (16). More solid data are needed in the future to corroborate the advantage of 7T for predicting IDH mutation in other sequences such as CEST.

Finally, we highlight that the achieved best prediction results in both single-modal and multi-modal Magnetic Resonance (MR) data are attributed to the advances of CNN model. Our CNN model, rather than radiomics-based methods, optimally utilizes the information from either CEST or T1 and further properly combines the information. From the methodological view point, the classical radiomics-based methods extract a set of handcrafted features from the tumor region and generate predictions based on the manually extracted feature and selected the conventional machine learning methods (logistic regression and SVM are used in this work). Such framework with separated feature extraction and model building could lead to limitation in either of the stages, indicated by the large variations under selections of different machine learning methods and their settings. On the other hand, the CNN automatically learns to extract abstract features from the data, where the feature extraction and classifier building are integrated and jointly optimizable (17). In addition, the CNN learns to get rid of redundant information and highlights different features among different imaging data at starting feature extraction stage, which greatly benefits multi-modal information fusion. The radiomics-based method extracts same set of features and then starts to reduce the redundant information at later classifier building stage, potentially bottlenecking the prediction performances. Using L1-penalty to select sparse features indeed significantly improved the prediction capacity of logistic regression, but still the final performance is not close to results from CNN. And from another perspective, the success of sparsity constraint on the radiomics features also indicates that a large part of the radiomics features extracted first stage is not beneficial to the prediction. Finally, the deep layer CNN has strong capacity of representation and could tackle complex non-linear mapping between the features and the prediction goals (18–20). The SVM model with non-linear kernels only fixes part of the issues but is still restricted by its simple mathematical form of the model. These advantages of CNN theoretically guarantee the improvements in the prediction.

When comparing and interpreting the prediction performances of the existing studies, a complication is raised by the differences of the definition of samples and validation schemes. For example, Calabrese et al. used multi-modal structural Magnetic Resonance (MR) data from each patient as an investigated sample and reported an AUC of 0.96 (21), while Chow et al. and Chang et al. used each axial slices from multi-modal structural Magnetic Resonance (MR) data as a sample and respectively reported AUCs of 0.86–0.96 and 0.93–0.95 (3, 22). However, it can be noted that the methods of Chow et al. and Chang et al. worked on the whole axial slice including the non-tumor regions, which could provide additional information. Indeed, when Chow et al. removed the non-tumor regions from the inputs, the performances of their method dropped to 0.81–0.88. Fukuma et al. conducted experiments using tumor region slices from T1 image and obtained AUC of 0.699 (23). Overall, we expect the performance of our method could be comparable and competitive to these when all conditions are aligned. As the first work studying CEST with CNN, our CNN model could be conventional and thus conservative when comparing our CNN design to other CNN-based studies (3, 21–23) In our design, the annotation mask from T1 image is utilized to enhance the predictive information and improves the prediction capacity, which is in line with the design in (3), which utilized ResNet as the CNN building blocks Aand combined CNN and radiomics. These advanced designs are expected to be adopted to our work and lead to potential improvements in future works.

There still exist several limitations of this study. Firstly, although the number of glioma cases included in this study is already the largest in the field of ultra-high field Magnetic Resonance (MR), it is still far from adequate for deep learning. Here we restricted the CNN to work on 2D slices to increase the sample size. We are still recruiting patients in our undergoing study, and the prospective clinical trial is conducted to further verify the model. Second, the present study failed to consider the spatially heterogeneous distribution of IDH mutation and instead treated each case as a whole as mutated or wild type. We are currently collecting multi-point biopsies to predict the spatial distribution of IDH mutation by point-to-point imaging analysis. Third, tumor grade between the two study groups may be a confounding factor to result (high grade glioma tends to a higher APT%), thus in the further study patients diagnosed as astrocytoma, aWHO grade 4, IDH mutant should be recruited to solidate the result. Fourth, a five pool Lorentzian model for Z-spectrum acquisition scheme and data evaluation protocol was used for CEST MRI, however in this article, we focused on APT-CEST as it has been shown to be the most sensitive contrast in detecting glioma histopathological characteristics. Nevertheless, we acknowledge that investigating other parameters can help confirm the value of metabolic information in glioma detection. In future research, we plan to evaluate other CEST parameters to gain a more comprehensive understanding of their potential clinical utility. Fifth, the automatic tumor segmentation from T1 image to generate the annotation mask was not integrated into the CNN framework, which may hinder the ease of its clinical application. Besides, since our CNN model was developed from T1 + CEST + annotations, other modalities such as DWI may be introduced to improve the reliability of the model in the future.

## Conclusions

We developed a deep learning model that can reliably predict the IDH status of gliomas based on conventional Magnetic Resonance (MR) and CEST imaging at 7.0 Tesla. 7T CEST and sMRI jointly offer improved sensitivity and specificity of preoperative non-invasive imaging for the diagnosis of IDH mutation status. As the first study of CNN model on imaging acquired at ultra-high field MR, our results could demonstrate the potential of combining ultra-high field CEST and CNN for developing an effective practical tool for the noninvasive characterization of gliomas to support individualized treatment planning.

## Data availability statement

The raw data supporting the conclusions of this article will be made available by the authors, without undue reservation.

## Ethics statement

The studies involving human participants were reviewed and approved by the Institutional Review Board of Huashan Hospital, Fudan University (protocol code: KY2020-675 and date of approval: 28 April 2020). The patients/participants provided their written informed consent to participate in this study.

## Author contributions

Conceptualization QY and ML. Methodology, YY and WY. Software, Y-HC, Y-CH and LAP. Validation, YY, JC and YFY. Formal analysis, YY and YFY. Investigation, YFY and JC. Resources, QY and ML. Data curation, YFY and Y-HC. Writing—original draft preparation, YFY, ML and QY. Writing—review and editing, ML and QY. Visualization, YFY, YY and Y-HC. Supervision, JC and QY. Funding acquisition, QY. All authors have read and agreed to the published version of the manuscript. All authors contributed to the article and approved the submitted version.
