# Supplementary material for: Convolutional neural network to predict IDH mutation status in glioma from chemical exchange saturation transfer imaging at 7 Tesla
Source: Front Oncol. 2023 May 8;13:1134626. doi: 10.3389/fonc.2023.1134626 (PMC10200907; doi:10.3389/fonc.2023.1134626)
Supplement: Supplementary file 1 [file DataSheet_1.docx]

**
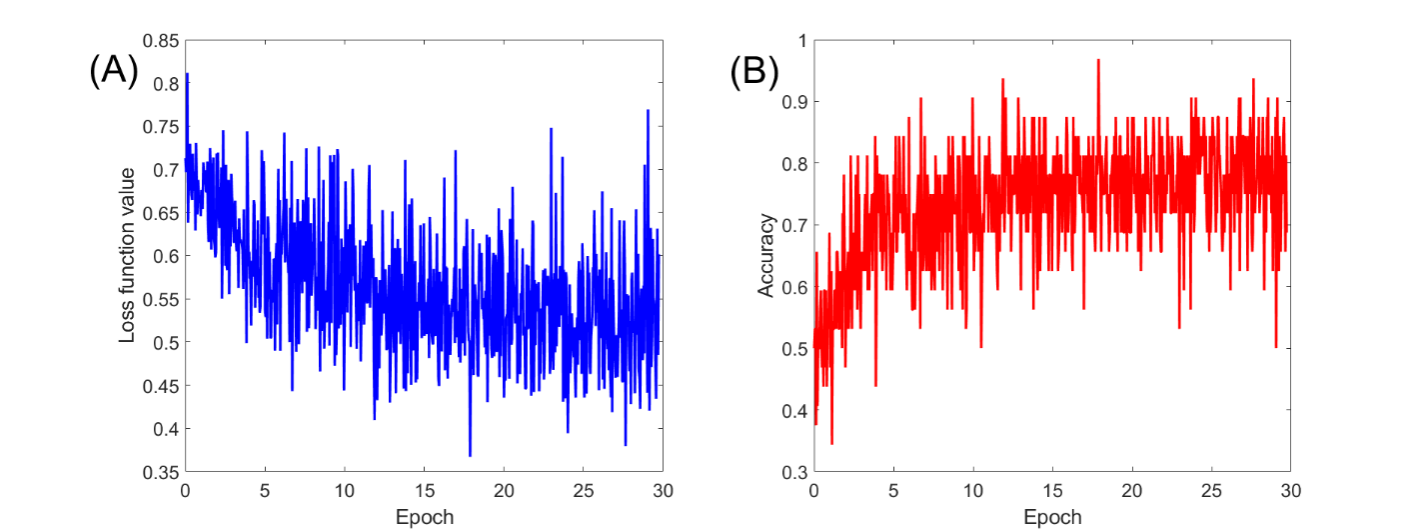
**

**Figure S1**. The curves for (A) loss function value and (B) accuracy in the training sets along with the training epochs, from one realization of the training as an example. Note that each training epoch contained 102 training iterations since the batch size is set to be 32.


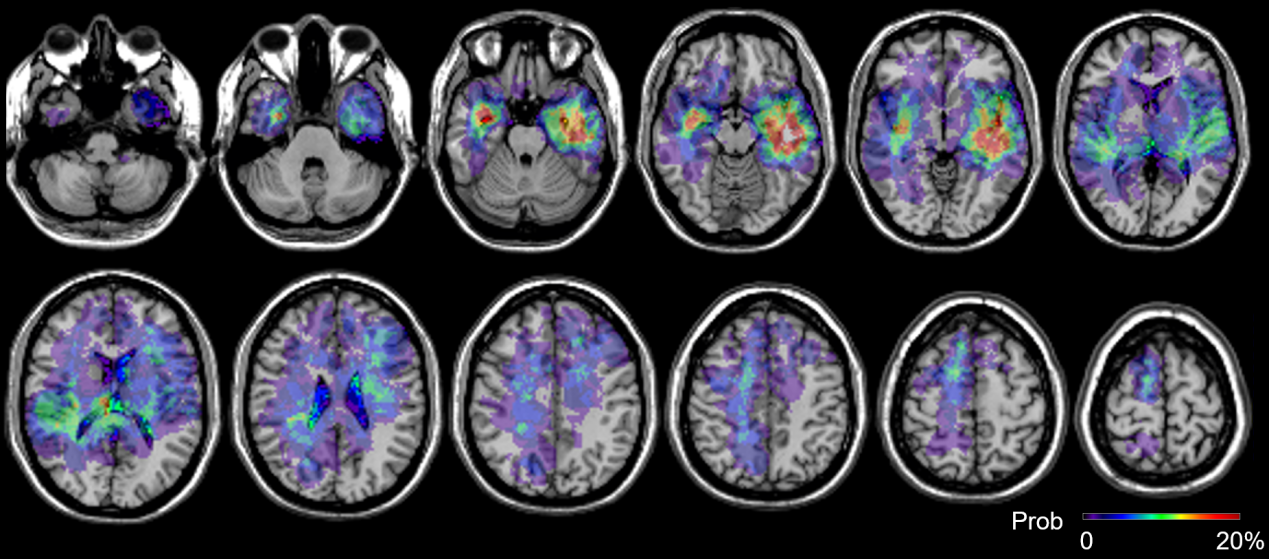


**Figure S2**. Spatial distribution of lesions included in this study and probability mapping (possibility from 0 to 0.2) was attached below.
