# Supplementary material for: Convolutional neural network to predict IDH mutation status in glioma from chemical exchange saturation transfer imaging at 7 Tesla
Source: Front Oncol. 2023 May 8;13:1134626. doi: 10.3389/fonc.2023.1134626 (PMC10200907; doi:10.3389/fonc.2023.1134626)
Supplement: Supplementary file 2 [file Table_1.docx]

| **Penalty** | **Input** | **ACC (%)** | **SEN (%)** | **SPE (%)** | **AUC** |
| --- | --- | --- | --- | --- | --- |
| L1 | T1+annotation | 67.36±1.70 | 67.16±2.35 | 67.57±1.81 | 0.6736±0.0170 |
|  | CEST+annotation | 72.03±1.37 | 77.51±3.82 | 66.89±2.68 | 0.7220±0.0151 |
|  | CEST+T1+annotation | 76.35±1.98 | 77.95±3.04 | 74.89±1.92 | 0.7641±0.0204 |
| L2 | T1+annotation | 59.83±1.62 | 53.34±3.12 | 65.93±1.14 | 0.5963±0.0155 |
|  | CEST+annotation | 63.18±1.04 | 78.14±2.73 | 49.29±3.06 | 0.6372±0.0120 |
|  | CEST+T1+annotation | 66.50±1.22 | 66.47±2.87 | 66.56±1.17 | 0.6652±0.0130 |
| Elasticnet | T1+annotation | 49.02±1.62 | 93.25±3.41 | 8.23±4.35 | 0.5074±0.0054 |
|  | CEST+annotation | 59.42±1.69 | 51.44±2.42 | 66.77±2.06 | 0.5911±0.0157 |
|  | CEST+T1+annotation | 63.82±0.10 | 76.28±2.53 | 52.28±1.30 | 0.6428±0.0100 |

**Table S1**. The prediction results using logistic regression with different penalty. L2-penalty and Elasticnet penalty (L1+L2, with ratio of 0.5) were tested.
