# Supplementary material for: Convolutional neural network to predict IDH mutation status in glioma from chemical exchange saturation transfer imaging at 7 Tesla
Source: Front Oncol. 2023 May 8;13:1134626. doi: 10.3389/fonc.2023.1134626 (PMC10200907; doi:10.3389/fonc.2023.1134626)
Supplement: Supplementary file 3 [file Table_2.docx]

| **Kernel** | **Input** | **ACC (%)** | **SEN (%)** | **SPE (%)** | **AUC** |
| --- | --- | --- | --- | --- | --- |
| Linear | T1+annotation | 49.85±5.05 | 55.27±8.51 | 44.70±16.99 | 0.4998±0.0436 |
|  | CEST+annotation | 51.47±2.61 | 71.84±24.82 | 32.5825.06 | 0.5221±0.0176 |
|  | CEST+T1+annotation | 46.59±4.76 | 66.20±11.81 | 28.19±3.02 | 0.4720±0.0475 |
| Polynomial | T1+annotation | 52.28±1.73 | 14.21±1.00 | 87.54±1.70 | 0.5087±0.0077 |
|  | CEST+annotation | 51.96±0.95 | 97.89±1.21 | 9.36±1.76 | 0.5363±0.0055 |
|  | CEST+T1+annotation | 55.48±3.57 | 59.98±28.06 | 52.07±30.20 | 0.5602±0.0273 |
| Radial | T1+annotation | 54.80±1.23 | 68.83±4.04 | 41.92±1.53 | 0.5537±0.0156 |
| Basis Function | CEST+annotation | 58.08±10.1 | 83.30±3.00 | 34.68±2.6 | 0.5899±0.0082 |
|  | CEST+T1+annotation | 64.13±1.91 | 66.30±2.61 | 62.11±1.73 | 0.6420±0.0194 |
|  | T1+annotation | 52.20±3.32 | 52.48±4.47 | 51.93±3.41 | 0.5221±0.0332 |
| Sigmoid | CEST+annotation | 52.07±1.41 | 52.32±1.99 | 51.81±1.73 | 0.5206±0.0142 |
|  | CEST+T1+annotation | 54.71±3.74 | 54.88±3.75 | 54.54±4.19 | 0.5471±0.0372 |

**Table S2**. The prediction results using SVM with different kernels. “Linear” refers to a linear SVM.
